# An Algorithmic Approach for the MIS Repair of Ventral Midline Hernias Associated With Diastasis of the Rectus Abdominis Muscle

**DOI:** 10.3389/jaws.2022.10864

**Published:** 2022-12-02

**Authors:** Cosman Camilo Mandujano, Diego L. Lima, Jason Xia, Prashanth Sreeramoju, Flavio Malcher

**Affiliations:** ^1^ Department of Surgery, Montefiore Medical Center, Bronx, NY, United States; ^2^ Abdominal Wall Reconstruction Program, Department of Surgery, Montefiore Medical Center, Bronx, NY, United States

**Keywords:** robotic abdominal wall repair, ventral hernia repair, laparoscopic ventral hernia repair, minimally invasive, diastasis of the rectus abdominis muscle

## Abstract

**Purpose:** We present our algorithmic approach for symptomatic ventral hernias with Diastasis of the Rectus Abdominis Muscle (DRAM).

**Methods:** Retrospective analysis of patients with symptomatic ventral hernias and DRAM undergoing hernia repair and plication of DRAM from July 2018–March 2021 was conducted. Based on our algorithm, patients were selected for an Endoscopic Onlay Repair (ENDOR) or a Robotic Extended Totally Extraperitoneal Ventral Repair (R-eTEP).

**Results:** We performed a R-eTEP in fifty-seven patients and an ENDOR in twenty-four patients. In the R-eTEP group, thirty-seven (65%) patients were female, the mean age was 54.8 (±10.6), and the mean BMI was 32 (±4.8). Fifty patients (87.7%) had multiple defects, of which 19 (38%) were recurrent hernias and 31 (62%) were incisional hernias. The mean operative time was 200 (±62.4) minutes, with two cases requiring a hybrid approach. The median length of stay was 1 day (0–12), and the median follow-up was 103 days. Twenty-four patients underwent an ENDOR, 19 females (79.2%), the mean age was 45.7 years (±11.7) and the mean BMI was 28 (±3.6). 13 patients had isolated umbilical or epigastric hernias. The mean operative time was 146.2 min (±51.1). Fibrin sealant and suture was the predominant method for mesh fixation, and most cases were performed in an ambulatory setting. Four patients developed post-operative seromas; one requiring drainage due to infection. The Median follow-up was 48.5 days (10–523), with two reported hernia recurrences.

**Conclusion:** An algorithmic approach for adequate patient selection was shown to be safe for treating ventral hernias with DRAM.

## Introduction

Diastasis of the Rectus Abdominus Muscle (DRAM) is a common abnormality due to increased abdominal pressures and or weakening of the linea alba resulting in a widening of the inter-rectus distance [1–3]. It is most commonly seen in females, obese patients, and individuals with prior abdominal surgeries [2–5]. The resulting midline bulge is associated with a negative body image, musculoskeletal pain, and occasionally urogynecological symptoms [3, 5].

Ventral hernias and concomitant symptomatic or asymptomatic DRAM are common. An untreated DRAM at the time of a ventral hernia repair has been associated with a higher risk of recurrence after ventral hernia repairs (VHR) [4, 6–8]. Therefore, we have tailored our approach based on the hypothesis that repairing an associated diastasis during a VHR may lead to a lower recurrence rate. The surgical management of ventral hernias is heterogeneous. The best location of the mesh and the surgical approach is still controversial.[9] Open, laparoscopic, and robotic approaches have been described in the literature.[9] However, the appropriate patient selection for the different operative approaches to treat this entity remains a subject of debate [10, 11].

The aim of this study is to present our algorithmic approach and early results for the treatment of symptomatic small ventral hernias with concomitant DRAM.

## Methods

### Study Design

This is a descriptive, retrospective study of consecutive patients with symptomatic ventral hernias and DRAM undergoing hernia repair and plication of the diastasis from July 2018–March 2021. Data were obtained from electronic health records from a single academic medical center. This study was approved by the Institution Review Board number (IRB # 2020-11160) and all Health Insurance Portability and Accountability Act (HIPPA) compliant mechanisms were followed.

### Inclusion Criteria

Adult male and female patients >18 years of age with a symptomatic single or multiple ventral hernias (<5 cm in size) undergoing elective minimally invasive VHR with an associated symptomatic or asymptomatic DRAM (Diastasis >2 cm as defined by the European Hernia Society, diagnosed clinically or *via* Computed Tomography).

### Exclusion Criteria

Patients <18 years of age, pregnant patients, emergency surgery, presence of a parastomal hernia, hernia size >5 cm, open surgery.

### Data Collection

Institutional Review Board approval was obtained to conduct this study. Data were retrospectively collected from a prospective database and divided into four sections: patient characteristics, Hernia characteristics, perioperative data, and patient outcomes. Patient demographics and comorbidities were analyzed: age, sex, body mass index (BMI), Diabetes Mellitus (DM), Hypertension, Chronic obstructive pulmonary disease (COPD), smoking status, Stroke, Cardiovascular accident (CVA), previous myocardial infarction, and American Society of Anesthesiologists (ASA) class. We did not collect data from open surgery as our objective was to show the early results of our minimally invasive procedures.

Preoperative data in the setting of incisional or recurrent hernias included Swiss cheese defects, type of primary hernia and or presence of multiple hernia defects.

Intraoperative and postoperative data consisted of the type of the approach, fixation of the mesh, duration of the surgery, length of stay (LOS), complications, readmissions, and follow-up.

### Statistical Analysis

Descriptive analysis was performed. Categorical variables are expressed as counts and percentages. Continuous variables were reported as mean and standard deviation for continuous variables whose distribution approximated normality and median and range for those with skewed distributions. Chi-square and Fisher’s Exact tests were used for categorical variables. T-tests and Wilcoxon rank-sum tests were used for continuous variables.

### Anatomy and Definition

Diastasis recti manifest as a midline abdominal bulge due to an attenuated linea alba with an increased laxity of the ventral abdominal wall musculature [1–3]. Anatomically, the linea alba’s width ranges from 11 to 21 mm between the xiphoid process and the umbilicus and decreases from 11 to 2 mm from the umbilicus to the pubic symphysis [5]. The thickness of the linea alba decreases towards the pubic symphysis; however, the posterior sheath is slightly thicker above the umbilicus compared to the anterior sheath [5]. The definition of DRAM varies in the literature, commonly defined as the distance from the muscular borders in the midline ranging from 2.2 to 3 cm above the umbilicus in a relaxed state, and can be classified as mild (<3 cm), moderate (3–5 cm), and severe (>5 cm) [7, 12]. Other classification systems such as the Nahas and Beer classification (which are based on the myofascial deformity, etiology, and the normal width of the linea alba in nulliparous women) have been utilized in the literature [5]. Most recently in 2021, the European Hernia Society (EHS) defined a rectus diastasis as a widening of the linea alba exceeding 2 cm and proposed a classification based on the width of muscle separation, post pregnancy status, and whether or not there is a concomitant hernia.[13].

### Indications for Surgery

The most common indication for surgical intervention in patients with DRAM is discomfort and cosmesis [5, 14]. There is not clear evidence that the presence of symptomatic or asymptomatic DRAM at the time of a ventral hernia repair is a risk factor for recurrence when left untreated, but in our institution it is part of our algorithmic approach [4, 6–8].

### Treatment Algorithm

Different options for treating midline hernias with symptomatic or asymptomatic DRAM exist, including non-operative management with core strengthening, aerobic activity, and neuromuscular reeducation for patients with minimal symptoms or not interested in undergoing surgical intervention [1, 2, 15–17].

For symptomatic small ventral midline hernias with associated symptomatic or asymptomatic DRAM, open and minimally invasive techniques either *via* laparoscopy or robotic surgery with or without the use of mesh are available [1, 2, 7, 18–21]. In our practice, we utilize mesh reinforcement for all of our patients and propose the following algorithm.

For patients with symptomatic umbilical hernias with DRAM requiring panniculectomy (excess skin flaps from weight loss or prior pregnancies, skin ulceration, previous scars), we perform an open approach with plication of the DRAM and hernia repair with onlay mesh.

If the patient has high cosmetic expectations, we refer our patients to the Plastic and Reconstructive surgery team to perform a conjoint abdominoplasty with the plication of the DRAM and hernia repair with onlay mesh. We have excluded patients undergoing open repair as the aim of this study is to describe our minimally invasive surgery (MIS) algorithmic approach.

For patients who do not require a panniculectomy, we perform an Endoscopic Onlay Repair (ENDOR) if the BMI is <30 mg/kg^2^. The rationale for not offering this approach for patients with BMI>30 is based on our published experience with a higher Surgical Site Ocurrences (SSO) rate in these patient populations.^7^ENDOR is the first choice for these patients as it offers easier ergonomics for traditional laparoscopic skills, allows anterior plication of the DRAM (preventing a cosmetically undesirable anterior ridge) and an onlay mesh positioning (maintaining a virgin retromuscular plane in case of a potential recurrence).

**FIGURE 1:**
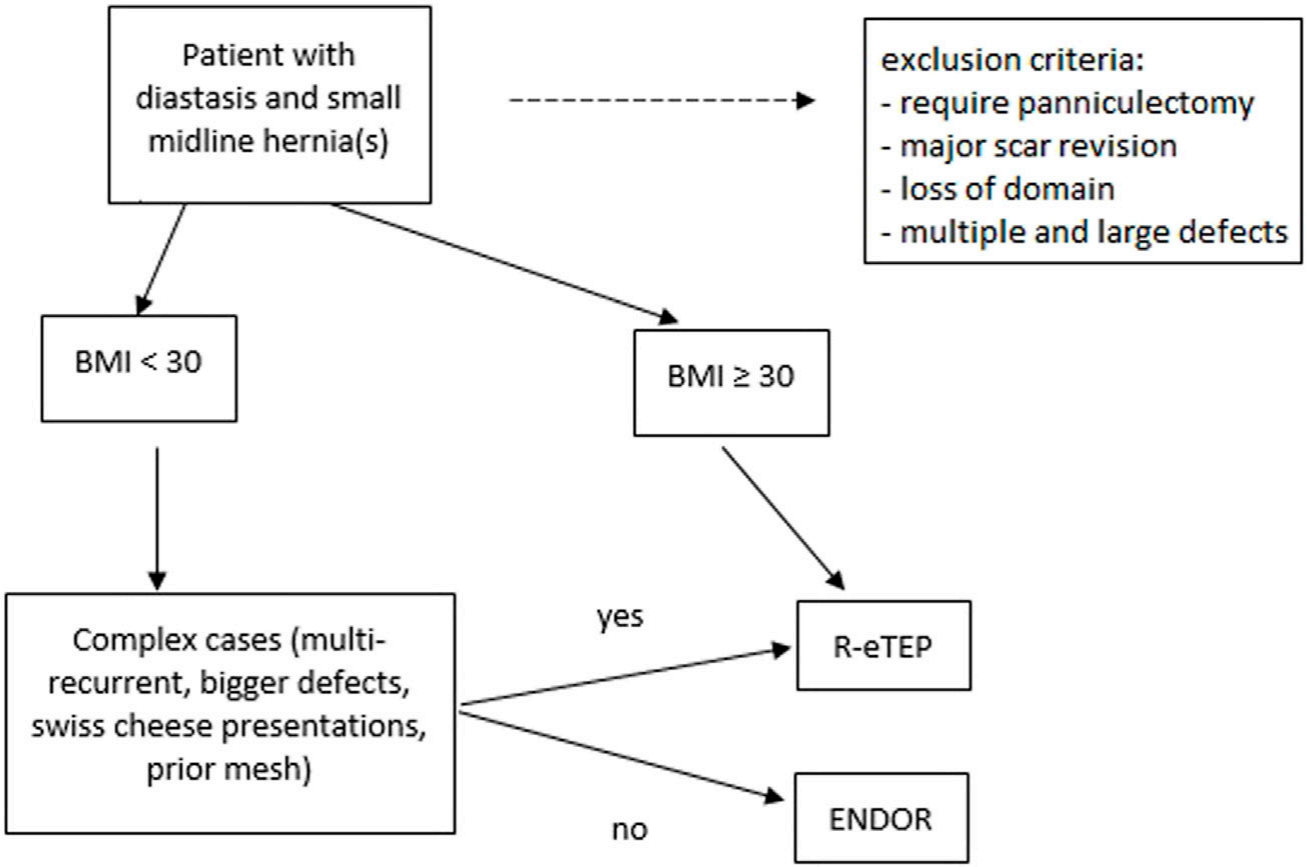
Algorithm to the treatment of ventral hernias associated to Diastasis of the Rectus.

For patients with symptomatic small ventral midline hernias with DRAM with BMI ≥30, we perform a Robotic Extended Totally Extraperitoneal repair (R-eTEP) with posterior plication of the DRAM, and hernia repair with sublay retromuscular mesh to decrease the wound morbidity of an ENDOR technique for these patients (Figure 1).

### Technique

#### Robotic extended totally extraperitoneal ventral hernia repair

The patient is placed in the supine position The patient´s hips are placed over the operating table’s flexion point. The bed is flexed, extending the working space between the subcostal margin and Anterior Superior Iliac Spine (ASIS) to create more space for our port placement. Next, upon preoperative review of cross-sectional CT imaging, the width of the retro rectus space is measured subsequently marked in the patient. A 5 mm Fios port (Applied Medical) is placed in the Left Upper Quadrant (LUQ) just medial to the lateral edge of the rectus muscle. The retro rectus space is identified under vision after traversing the anterior sheath and rectus muscle, then the port is directed inferiorly at a 45-degree angle, and insufflation is initiated. Blunt dissection is performed to allow space for our second port placement 8 cm below the LUQ port 1 cm medial to the semilunar line to avoid any injuries to the neurovascular bundles. A spinal needle is utilized to ensure a safe tract into the retro rectus space, and an 8 mm robotic port is placed under direct vision. Electrocautery with hook or Maryland dissector is used to create space inferiorly for an additional 8 mm robotic port at the Left Lower Quadrant (LLQ), 8 cm inferiorly. At this time, the camera is switched to the inferior port to complete our dissection superiorly, providing good exposure prior to docking the robot and the initial 5 mm optical trocar is exchanged for an 8 mm robotic port. The robot is docked from the right side of the patient. We initiate our dissection lateral to medial towards the linea alba performing a cross over at the epigastric area, taking advantage of the preperitoneal fat tissue of the round ligament of the liver, into the contralateral retro rectus space starting distally from the defect progressing towards the hernia, identifying the hernia sac and reducing its contents into the abdominal cavity. After our dissection is complete, we measure the defect’s length and width, including the DRAM, ensuring 3–5 cm overlap. At this point, any opening on the peritoneum or posterior fascia is closed using running 3–0 barbed slowly absorbable sutures. We, then, plicate the DRAM, including the hernia defect’s closure with a running 0 barbed slowly absorbable 180 sutures. We then transition to laparoscopy to introduce the mesh, fixed in two points (suprapubic and subxiphoid) with 0 vicryl^®^ trans fascial sutures to help positioning; once the mesh lays flat in the retro rectus space, we proceed to deflate the abdomen completing the procedure. Drains are not routinely used.

#### Endoscopic Onlay Repair

The patient is placed supine under general anesthesia, with a slight extension of the hip and the legs abducted. The surgeon is positioned between the patient’s legs and the assistant laterally.

A 2 cm transverse incision just above the pubis is performed, followed by subcutaneous dissection exposing the rectus abdominis muscle’s anterior aponeurosis. The subcutaneous tissue is separated from the anterior aponeurosis with monopolar cautery both superior and laterally to create sufficient space for the placement of a 12 mm camera port and two 5 mm working ports bilaterally. A purse-string suture is performed in the suprapubic incision to secure the camera port and prevent CO_2_ leakage. The subcutaneous tissue is dissected off the rectus muscle’s anterior aponeurosis with electrocautery maintaining a CO_2_ insufflation at 8–10 mmHg. The umbilicus is disinserted from the aponeurotic muscle plane, and the dissection is extended superiorly to the xiphoid process and 12–15 cm laterally towards the ribs. The hernia sac is dissected, and the contents are reduced to the abdominal cavity. In our experience, if a peritoneal violation occurs, we have not encountered a limited exposure to the operative field. Next, we plicate the DRAM with a running suture to approximate the edges of the rectus muscles with 0 barbed suture 180. The suture line extends from the xiphoid to at least 2–3 cm below the umbilicus. A polypropylene or monofilament polyester mesh is introduced in the craniocaudal direction from the xiphoid to the 3–4 cm below the umbilicus with a lateral overlap of at least 3–5 cm. The mesh may be self-gripping or can be secured with tackers, suture, or glue. The umbilical stalk is fixated back to the musculoaponeurotic plane through one or two simple sutures, and a closed drain suction is introduced *via* a lateral port to prevent seroma formation.

## Results

A total of 81 patients were included on the analysis. A R-eTEP was performed in Fifty-seven patients, and twenty-four underwent an ENDOR technique. Patients’ demographics are as listed in Table 1.

**TABLE 1:** Patient demographics.

	eTEP (*n* = 57)	ENDOR (*n* = 24)
	n (%)	n (%)
Sex		
Female	37 (64.9)	19 (79.2)
Male	20 (35.1)	5 (20.8)
Mean age, years (range)	54.8 (34–80)	45.7 (27–64)
Mean BMI, kg/m^2^ (±SD)	32 (4.8)	28 (3.6)
ASA		
I	1 (2)	9 (37.5)
II	29 (51)	15 (62.5)
III	26 (45)	0
IV	1 (2)	0
Hypertension	27	5
Diabetes Mellitus	15	3
Hypercholesterolemia	23	3
Smoking	6 (10.5)	1 (4.2)
Former smoker	18 (31.6)	0
COPD	3 (5.3)	0
CAD	3 (5.3)	0
Stroke/CVA	2 (3.5)	0
MI	1 (2)	0
CKD	2 (3.5)	0

eTEP, extended totally extraperitoneal; ENDOR, endoscopic onlay repair; BMI, body mass index (kg/m^2^); ASA, American society of anesthesiologists physical status classification; COPD, chronic obstructive pulmonary disease; CAD, coronary artery disease; CVA, cerebrovascular accident; MI, myocardial infarct; CKD, chronic kidney disease.

### R-eTEP Cases

Thirty-seven (65%) patients were female, the mean age was 54.8 (±10.6), and the mean BMI was 32 (±4.8) (Table 1). Hernia characteristics are as listed in Table 2. Fifty patients (87.7%) had multiple defects, nineteen (38%) had a recurrent hernia, and thirty-one (62%) presented an incisional hernia. Mean defect width was 4.3 cm (±1.9) and mean mesh area 507.4 cm^2^ (±128.2) (Table 2).

**TABLE 2:** Hernia types.

	eTEP (*n* = 57)	ENDOR (*n* = 24)
	n (%)	n (%)
Isolated Umbilical (M3)	5 (8.8)	11 (45.8)
Isolated Epigastric (M2)	2 (3.5)	2 (8.4)
Multiple defects (M2/M3)	50 (87.7)	11 (45.8)
Recurrent hernia	19 (38.0)	8 (72.7)
Incisional	31 (62.0)	3 (27.3)

eTEP, extended totally extraperitoneal.

ENDOR, endoscopic onlay repair.

M2/M3, European Hernia Classification of Ventral Hernias.

All patients underwent a robotic approach, and the perioperative outcomes are listed in Table 2. The mean operative time was 200 (±62.4) minutes. The hernia defects were closed in all patients, and the mesh was secured with cardinal sutures in most cases (72%). We utilized fibrin sealant in conjunction with cardinal sutures in five cases. Two patients were converted to a hybrid operation. One case presented extensive fibrosis from a prior procedure leading to a challenging exposure and the second case was due to an incarcerated bowel who ultimately needed to undergo a segmental resection. Patients’ outcomes are as listed in Table 3.

**TABLE 3:** Perioperative results.

	eTEP (*n* = 57)	ENDOR (*n* = 24)
	n (%)	n (%)
Surgical Approach		
Robotic	57 (100)	3 (12.5)
Laparoscopic	0	21 (87.5)
Mean Surgical time (min), (SD)	200 (±62.4)^a^	146.2 (±51.1)^a^
Median Estimated blood loss (ml)	20 (0–300)^b^	10 (5–40)^b^
Median defect area (cm^2^)	30 (2–570)^b^	4 (1–81)^b^
Mean defect width (cm)	4.3 (±1.9)^a^	2.2 (±1.6)^b^
Mean mesh area (cm^2^)	507.4 (±128.2)^a^	317.1 (±119.2)^a^
Mesh fixation		
None	1 (2)	1 (4.2)
Tacks	3 (5)	2 (8.4)
Suture	41 (72)	1 (4)
Fibrin glue alone	0	7 (29.2)
Fibrin glue and suture	4 (7)	13 (54.2)
Fibrin glue and tacks	1 (2)	0
Suture and tacks	7 (12)	0
Median Length of Stay (days)	1 (0–12)^b^	0 (0–3)^b^
Conversion to open	2 (3.5)	0
Intraoperative complication		
None	53 (93)	23 (95.8)
Serosal tear	2 (3.6)	0
Bleeding	1 (1.7)	1 (4.2)
Enterotomy	1 (1.7)	0

eTEP, extended totally extraperitoneal.

ENDOR, endoscopic onlay repair.

^a^ Standard Deviation.

^b^ Range.

The median LOS was 1 day (0–12), and the median follow-up was 103 days (10–713). Five patients developed post-operative seromas, and one patient developed a post-operative hematoma, which all resolved spontaneously (Table 4).

**TABLE 4:** Postoperative outcomes.

	eTEP (*n* = 57)	ENDOR (*n* = 24)
	n (%)	n (%)
Post-operative complications		
No	51 (89.5)	19 (79.2)
Seroma	5 (8.8)	4 (16.6)
Hematoma	1 (1.7)	0
SSI	0	1 (4.2)
Hernia Recurrence	0	2 (8.3)
30 days readmission	0	1 (4.2)
Follow-up	54 (94.7)	24 (100)
Median follow-up, days (range)	103 (10–713)^a^	48.5 (10–523)^a^

eTEP, extended totally extraperitoneal.

ENDOR, endoscopic onlay repair.

^a^ Range

### ENDOR Cases

Twenty-four patients underwent an ENDOR approach. Nineteen patients were female (79.2%), mean age was 45.7 years (±11.7), with a mean BMI of 28 (±3.6) (Table 1). Thirteen patients had isolated umbilical or epigastric hernias (Table 2). Three patients (12.5%) were submitted to a robotic approach. Mean defect width was 2.2 cm (±1.6) and mean mesh area was 317.1 (±119.2) (Table 2). The mean operative time was 146.2 min (±51.1). Mesh was predominantly fixated with a fibrin sealant and suture. There was no conversion to open surgery or from robotic to laparoscopic surgery. Only one patient had intraoperative bleeding due to a tacker, which was not significant. Most patients underwent same day of surgery, with our longest LOS reported of 3 days (Table 2). Four patients developed post-operative seromas, one requiring readmission and drainage due to infection. The majority of patients (87.5%) had a subcutaneous drain placed, which was removed during the first post-operative office visit 2 weeks after the surgery, or when the output was less than 50 cc/day. The Median follow-up was 48.5 days (10–523), and two patients developed a hernia recurrence (Table 4). One patient was a heavy smoker that refused to stop smoking and the other patient did not take care of her drain, developing an infected seroma treated with percutaneous drainage and IV antibiotics.

## Discussion

The treatment of small ventral hernias with concomitant DRAM remains a subject of debate with no clear guidelines or quality evidence to support an optimal approach when these entities coincide [10, 11]. As the field of hernia surgery continues to evolve with the expansion of MIS approaches, several techniques have been described in the literature for the management of ventral hernias with DRAM [7, 8]. Surgical correction of DRAM follows two main trends, plication versus midline mesh reinforcement and both methods seem to be safe, with high patient satisfaction in support of the correction of this entity [1, 14, 22, 23]. More importantly, failure to correct a DRAM at the time of ventral hernia repair is associated with higher recurrence rates which demands attention and correction when both entities are present.

In our practice, we incorporate plication and mesh reinforcement, and we stratify our patients to an MIS or open repair with an algorithmic approach with the aim of achieving an individualized and optimal cosmetic and functional outcome. In our experience, most of our patients are mainly driven by the functional limitations elicited by their symptomatic hernias, therefore we have noticed an inclination towards MIS interventions where an abdominoplasty with the assistance of the plastic surgery team is not performed, and therefore we have excluded these cohort of patients from our results.

This MIS approach performing a subcutaneous dissection above the anterior rectus sheath has been described by different authors with several modifications [7, 8, 18, 19, 24] to this technique and we refer to it as ENDOR (Endoscopic Onlay Repair) in an attempt to utilize a standardized term. In regard to our technique, we perform an ENDOR for patients with BMI<30 to decrease the associated wound morbidities that we noticed from our initial experience with this approach. The patients selected for ENDOR are healthier patients with smaller defects, however we limit our exclusion criteria to BMI>30 rather than patient specific comorbidities or hernia size defect. The patient’s characteristics and hernia defect size remained homogeneous in our expanded series, in patients who underwent and ENDOR approach. Since our initial report in 2020, we have expanded our series to 24 patients with similar outcomes reporting a 16.6% rate of postoperative seromas compared to 18% from our initial experience and no additional recurrences (2%) from our initial series [7].

The evidence supporting minimally invasive approaches for ventral hernia repairs is well established in the literature, and the application of the robotic platform for complex abdominal wall reconstruction has grown over the past years [9]. The general principles in hernia repair of mesh utilization, access to the retro muscular space, primary fascial closure of the hernia defects and a MIS approach are all achieved with the application of robotic surgery [9]. The known benefits of decreased recurrence with mesh placement in the retro muscular space compared to Onlay, inlay or Sublay techniques as well as the lower incidence of surgical site infections supports our choice to perform robotic eTEP for patients with higher BMIs in the setting of small ventral hernias with associated DRAM [9].

While we support the use of the robotic platform for these cases, one of the limitations to this approach is the availability and surgeon’s proficiency with the utilization of the robot which may limit the application of this algorithmic pathway. However, it is worth mentioning that retro muscular access *via* a laparoscopic approach is also feasible which may permit the adoption of this pathway in the absence of the robotic Platform [23]. In 2019, Lu et al, performed a comparative review of outcomes for laparoscopic versus Robotic ETEPs with comparable outcomes between the two [25]. However, we believe there is a benefit to the use of the robotic platform particularly for higher BMI patients and larger defects where laparoscopy may be challenging. From a cost benefit standpoint, Warren et al. compared the direct hospital cost between laparoscopic IPOM vs. robotic retro muscular repairs and found no statistically significant difference.[26].

In our study, as expected in our algorithm, patients submitted to R-eTEP were older with more comorbidities and higher BMI. Furthermore, they had more incisional hernias and multiple defects than patients submitted to ENDOR. The patients included on the R-eTEP group included the small ventral midline hernias with DRAM and a broader spectrum of presentations with multiple midline hernias, recurrent and incisional, always associated with DRAM. Additionally, patients with BMI <30 and multiple defects such as inguinal hernias associated to ventral hernias, were also submitted to a R-eTEP procedure, which adds to the advantages of accessing the retromuscular space in these scenarios. That factor explains the higher complexity of demographics and presentations. Interestingly, despite a more complex cohort of patients, in our experience, we did not have recurrences in patients submitted to a R-eTEP so far. While it appears that a R-eTEP confers better post-operative outcomes regarding seroma formation and recurrences when compared to an ENDOR when evaluated individually, we have to consider that a larger sample size in the ENDOR group may likely yield comparable results in regards to seroma formation. In addition, none of the post-operative seromas required interventions; therefore, we still advocate for an ENDOR in patients with lower BMI. Overall, our short-term follow-up limits our ability to evaluate long-term outcomes regarding recurrences for both groups and will be a topic for evaluation in our long-term follow-up studies.

R-eTEP is not the first option for all patients. This technique requires the robotic platform, which is not always available, has a high operative time, and is technically more challenging. It explores the retrorectus space, which would preclude re-approaching this space in the event of a recurrence and disrupts the linea alba due to the transection of the posterior rectus sheath. On the other hand, ENDOR is technically easier and does not use the retrorectus space. The main disadvantage is the seroma formation as reported by several authors [25, 18, 7]. Both techniques avoid intraperitoneal mesh with its possible complications and the need for extensive fixation with tackers or sutures which can lead to acute or chronic pain.

### Limitations

Our study has several limitations. First, it is a retrospective, single-center study with a small sample. Data is also limited to short-term outcomes in the current study. Long-term variables, including chronic pain, quality of life measures, and aesthetic outcomes after repair of the diastasis, are essential to consider in future studies. Furthermore, information regarding long-term follow-up and recurrence rates is necessary to determine the effectiveness of repairing a diastasis in reducing recurrence rates in VHR. Finally, a cost analysis evaluation was not available.

## Conclusion

An algorithmic approach for adequate patient selection was shown to be safe for treating ventral hernias with concomitant DRAM.

## Data Availability

The raw data supporting the conclusion of this article will be made available by the authors, without undue reservation.

## Abbreviations

DRAM, diastasis of the rectus abdominis muscle; ENDOR, endoscopic onlay repair; R-eTEP, robotic extended totally extraperitoneal ventral repair; BMI, body mass index; VHR, ventral hernia repair; IRB, institution review board; HIPPA, The Health Insurance Portability and Accountability Act; IR, institution review; DM, Diabetes Mellitus; COPD, Hypertension, Chronic obstructive pulmonary disease; CVA, cerebrovascular accident; ASA, American Society of Anesthesiologists; LOS, length of stay; SSO, surgical site occurrences; MIS, minimally invasive surgery; CO_2_, carbon dioxide; LUQ, left upper quadrant; LLQ, left lower quadrant; IPOM, intraperitoneal onlay mesh.
